# Adult attachment style and cortisol responses across the day in older adults

**DOI:** 10.1111/psyp.12075

**Published:** 2013-06-30

**Authors:** Tara Kidd, Mark Hamer, Andrew Steptoe

**Affiliations:** Psychobiology Unit, Department of Epidemiology and Public Health, University College LondonLondon, England

**Keywords:** HPA axis, Cortisol, Adult attachment style, Whitehall II

## Abstract

The association between cortisol and adult attachment style, an important indicator of social relationships, has been relatively unexplored. Previous research has examined adult attachment and acute cortisol responses to stress in the laboratory, but less is known about cortisol levels in everyday life. The present study examined adult romantic attachment style and cortisol responses across the day. Salivary cortisol was collected at six time points during the course of the day in 1,807 healthy men and women from a subsample of the Whitehall II cohort. Significant associations were found between attachment on cortisol across the day and slope of cortisol decline. The lowest cortisol output was associated with fearful attachment, with preoccupied attachment having the highest levels and a flatter cortisol profile. The results tentatively support the proposition that attachment style may contribute to HPA dysregulation.

Attachment theory has been increasingly applied to understanding the development and progression of disease. In particular, it has been posited that insecure attachment could be a risk factor for a variety of health conditions (McWilliams & Bailey, 2010). Research has begun to focus on psychobiological pathways through which attachment style may confer increased risk, specifically the hypothalamic pituitary adrenal (HPA) axis. This paper aims to expand on previous work by examining adult attachment style and cortisol response across the day in healthy older adults.

The attachment system was originally conceived as a psychobiological process thought to ensure the survival of an infant by reinforcing basic capacities to respond to danger or potential threat (Bowlby, 1969). In human infants, physiological arousal is regulated by the response of the caregiver during a threat. The quality of these interactions is believed to be crucial in the development of brain regions associated with the regulation of stress systems (Teicher et al., 2002). Infants who do not receive adequate care are believed to have impaired stress regulation in adulthood and believe that others will not be available to alleviate their distress as a consequence of these early interactions (Main, 1990).

Adult attachment is described in terms of two independent dimensions, namely, attachment anxiety and avoidance (Brennan, Clark, & Shaver, 1998). Individuals who are high in attachment anxiety tend to maximize negative experiences and are hypervigilant to potential threat. Individuals high in attachment avoidance tend to minimize feelings of distress and direct attention away from potential threat by maximizing autonomous behavior strategies. By combining these two dimensions, four prototypic attachment styles can be produced consisting of secure attachment (low anxiety/low avoidance) and three insecure styles of preoccupied (high anxiety/low avoidance), fearful (high anxiety/high avoidance), and dismissive (low anxiety/high avoidance) (Bartholomew & Horowitz, 1991; Kidd, Hamer, & Steptoe, 2011).

Research has shown that there is a robust association between adult attachment and self-reported distress, including perceived stress and symptom reports, with those who are high in anxious attachment reporting greater distress (Kidd & Sheffield, 2005). In addition, attachment has also been linked to objective health outcomes (McWilliams & Bailey, 2010); however, the mechanisms underlying this association are less well understood. One pathway through which attachment may confer a risk to health is through the HPA axis. The HPA axis has been established as a mechanism through which the stress response may influence health outcomes, particularly through alterations in cortisol production (Kumari, Shipley, Stafford, & Kivimaki, 2011). It is perhaps especially pertinent to attachment as it has been shown to be activated during socioevaluative and interpersonal threat (Dickerson & Kemeny, 2004).

The cortisol response is often assessed in the laboratory, comparing stress reactivity between individuals with different characteristics. Several studies have reported that insecure attachment is associated with hyperreactive strategies, including increased reporting of perceived stress and cortisol response during acute stress in younger populations (Dewitte, De Houwer, Goubert, & Buysse, 2010; Powers, Pietromonaco, Gunlicks, & Sayer, 2006; Quirin, Pruessner, & Kuhl, 2008). Interestingly, these studies have shown that there appear to be gender and possible age effects. In regards to gender, there is a trend towards attachment avoidance in females and attachment anxiety in males, both being associated with increased cortisol reactivity to a stressor (Dewitte et al., 2010; Powers et al., 2006). Existing work has focused on younger adults and has reported increased cortisol response to an acute stressor for those who are anxiously attached (Dewitte et al., 2010; Powers et al., 2006; Quirin et al., 2008). In contrast, older age has been associated with hyporeactive responses in fearfully attached (high anxiety/high avoidance) older adults, despite their reporting high levels of perceived stress (Kidd et al., 2011). At present, this is the only study to our knowledge that has examined older age effects alongside attachment and cortisol response to acute stress.

Studying cortisol responses to laboratory stress is not without limitations; namely, it involves assessing acute responses to arbitrary short-term behavioral stimuli under artificial conditions that are seldom encountered in everyday life. It cannot be ruled out that the laboratory setting itself may exacerbate stress-related cortisol changes in individuals not able to control their responses using habitual attachment regulatory (e.g., hyperactivating or deactivating) strategies (Dewitte et al., 2010). Additional factors may also contribute to individual variation in response to a stressful experience in the laboratory setting, such as age and gender (Adam & Kumari, 2009). Past research has shown that men and women differ in the contexts in which they show increased HPA responses (Stroud, Salovey, & Epel, 2002). Although activation of the attachment system is believed to be similar for men and women, it appears that context (or, for our purposes, type of stress task) may influence when differences become apparent (Hicks & Diamond, 2011).

There has been little investigation into adult attachment and diurnal cortisol pattern to date. The possible influence of gender has been investigated in two studies only, with most focusing on young adult males or females. Adam and Gunnar (2001) found a negative relationship between attachment anxiety and baseline cortisol levels in females. Negative relationships have also been reported between attachment anxiety and the cortisol awakening response (CAR) (Hicks & Diamond, 2011; Quirin et al., 2008). Quirin et al. (2008) proposed that females high in attachment anxiety had an attenuated CAR as a consequence of elevated waking cortisol levels. Rifkin-Graboi (2008) examined both CAR and cortisol response across the day in young adult males, but only found an association between attachment anxiety and cortisol during the afternoon. Studies that have examined gender difference have reported mixed results. Hicks and Diamond (2011) found that only females high in attachment anxiety had a blunted CAR following a relationship dispute the previous evening. More recently, Jaremka et al. (2013) found that anxious attachment predicted higher cortisol levels across the day following discussion of marital problems. No interaction was found between attachment and gender on any of the cortisol outcome measures. Like the research on acute stress, these studies were conducted on relatively small samples of younger adults.

In this report, we analyzed a large sample of older men and women from the Whitehall II study, and tested the following hypotheses. First, individuals high in attachment anxiety (preoccupied and fearful) will report greater levels of perceived stress in comparison to those low in anxiety (secure and dismissive) during the course of the day. Second, individuals high in attachment anxiety will have an attenuated CAR compared with other attachment groups. Third, based on our previous study, we believe that a lower total cortisol output over the day will be associated with fearful attachment (high anxiety/high avoidance). Fourth, as no one has yet examined attachment and cortisol slope over the day, we hypothesized that one or more of the insecure attachment styles would be associated with flatter slopes of cortisol over the day. Finally, we examined the influence of gender on cortisol outcomes in this older population. We made no specific predictions regarding gender due to the array of findings in the literature.

## Method

### Participants

Analyses were carried out on a subsample of participants in the Whitehall II study, an epidemiological study of socioeconomic, psychosocial, and biological risk factors for coronary heart disease and other disorders of aging (Marmot & Brunner, 2005). Cortisol was collected over the course of the day during Phase 7 of the study (2002–2004). A total of 2,729 participants completed both the attachment measure in Phase 5 and returned saliva samples in Phase 7. Inclusion criteria for these analyses included no history or objective signs of coronary heart disease, hypertension, or inflammatory disease, no history of mental illness, or use of any steroid medication. Only those with waking cortisol samples taken within 10 min of reported waking time were included (Dockray, Bhattacharyya, Molloy, & Steptoe, 2008). After applying our inclusion criteria, 1,807 participants were eligible to take part in the study (1,407 males, 400 females; age range 50–73 years). All procedures were carried out with the written consent of the participants. These data were drawn from a different subset of Whitehall II participants from those in our previous laboratory study (Kidd et al., 2011). Ethical approval for the study was obtained from the University College London Medical School committee on the ethics of human research.

### Cortisol Collection and Analysis

The collection of cortisol has been described previously (Kumari et al., 2010). Briefly, participants were asked to provide six saliva samples in Salivettes over the course of a normal weekday at waking, at waking plus 30 min, 2.5 h, 8 h, 12 h, and at bedtime. Participants were instructed not to brush their teeth or to eat or drink anything for 15 min prior to sample collection. Participants were instructed to take the first sample “as soon as you open your eyes and before your feet touch the ground.” Salivary cortisol was measured using a commercial immunoassay with chemiluminescence detection (CLIA; IBL-Hamburg, Hamburg, Germany). The lower concentration limit of this assay is 0.44 nmol/liter; intraassay and interassay coefficients of variance were < 8%. Any sample over 50 nmol/liter was repeated.

Participants recorded information in a booklet on time of waking, time of day they had taken each sample, and if they had any caffeinated drinks, alcohol, or food. Participants also rated stress/worry for the previous 20 min to each of the saliva samples on a 5-point rating scale (ranging from 1 = *low stress*, to 5 = *high stress*). Stress scores were aggregated and averaged to give one total stress score. High scores indicate higher levels of perceived stress over the day.

### Questionnaires

The Relationship Questionnaire (RQ) is a single-item measure made up of four short paragraphs. Each paragraph describes a prototypical pattern of attachment behavior for adult romantic relationships (Bartholomew & Horowitz, 1991). Participants are asked to rate out of 100 their degree of compatibility for each paragraph. A score of 100 indicates that “the statement describes me exactly.” These ratings provide a continuous profile of an individual's attachment behavior. Participants were instructed to provide a different score for each paragraph. The statements are:

It is easy for me to become emotionally close to others. I am comfortable depending on them and having them depend on me. I don't worry about being alone or having others not accept me. *(Secure)*

I am uncomfortable getting close to others. I want emotionally close relationships, but I find it difficult to trust others completely, or to depend on them. I worry that I will be hurt if I allow myself to become too close to others. *(Fearful)*

I want to be completely emotionally intimate with others, but I often find that others are reluctant to get as close as I would like. I am uncomfortable being without close relationships, but I sometimes worry that others don't value me as much as I value them. *(Preoccupied)*

I am comfortable without close emotional relationships. It is very important to me to feel independent and self-sufficient, and I prefer not to depend on others or have others depend on me. *(Dismissive)*

The continuous scores on the four attachment items were used in the primary analyses of cortisol. But in order to characterize participants in terms of their attachment prototype, each individual was also classified into one of four groups, based on the highest score on these items. If the highest rating was tied between two paragraphs, participants were excluded from this categorization. This measure was administered in Phase 5 (1997–1999) of the Whitehall II study (Bartley, Head, & Stansfeld, 2007). The RQ also has been found to have good convergent and discriminant validity (Brennan et al., 1998) and reliability (Hazan & Shaver, 1987).

### Cortisol Data Reduction

The CAR was calculated by subtracting cortisol measured at time 1 (waking) from cortisol measured at time 2 (waking + 30 min). The pattern of cortisol over the day was analyzed by computing cortisol area under the curve with respect to ground (AUC_ground_) for the complete day session. AUC was calculated using the procedures described by Pruessner, Kirschbaum, Meinlschmidt, and Hellhamer (2003). The cortisol slope of change over the day was computed by regressing values on time, excluding the waking + 30 value. We calculated the difference in cortisol between each sample as a change per minute, and aggregated these values. High values indicate a more rapid decline in cortisol levels, whereas lower slope values reflect flatter diurnal rhythms.

### Statistical Analysis

Associations with the full range of scores on each attachment style and cortisol were analyzed using multiple linear regressions, entering ratings on the four scales simultaneously into the regression models for each cortisol parameter. Age, gender, body mass index (BMI), smoking status, waking time, employment grade (higher, intermediate, lower), and subjective stress were entered as covariates for all the analyses. These factors were included since they are known to be associated with cortisol (Adam & Kumari, 2009). Adjusted *R*^2^ and standardized beta values are reported throughout. Preliminary analyses were conducted to ensure no violations of the assumption of normality, linearity, multicollinearity, and homoscedasticity.

## Results

### Demographics

Participant characteristics are detailed in Table 1. Overall, a total of 1,807 participants were included in the study, with ages ranging from 45 years to 67 years. Although the primary analyses were based on continuous scores on the four attachment items, it is interesting that 747 participants were classed as having a predominantly secure attachment style, 262 fearful, 134 preoccupied, and 664 a dismissive attachment style. No individuals were excluded from this categorization on the basis of having tied scores on their preferred attachment style. There were no significant differences between attachments styles on gender, employment grade, BMI, smoking behavior, or time of waking (*p* >. 05). There was a significant difference between groups on age, *F*(3,1803) = 6.347, *p* =. 001, with those classed as fearful being younger than those with a secure (*p* =. 015) or dismissive attachment style (*p* <. 001).

**Table 1:** Characteristics of the Study Population

	Secure	Fearful	Preoccupied	Dismissive
	(*n* = 747)	(*n* = 262)	(*n* = 134)	(*n* = 664)
Age (years)	60.15 ± 5.77	58.95 ± 5.36	60.07 ± 5.68	60.71 ± 5.61
Smoking status (current)	75 (9.7%)	18 (6.7%)	16 (11.6%)	67 (9.7%)
BMI (kg/m2)	26.23 ± 4.06	25.72 ± 3.99	26.52 ± 4.37	26.10 ± 3.91
Gender (m/f)	594/153	200/62	104/30	560/104
Paid employment	176	57	32	155
Wake-up time (*SD* min)	6.42 ± 0:57	6.43 ± 67	6.45 ± 60	6.43 ± 63
Subjective stress (mean *SD*)	1.30 ± 0.07	2.13 ± 0.12	2.28 ± 0.17	1.38 ± 0.07

### Attachment Ratings and Perceptions of Stress

The association between attachment ratings and perceptions of stress were examined using the mean stress score. The model consisted of the four attachment styles and the covariates of age, gender, BMI, and smoking. Attachment did significantly predict the mean level of perceived stress over the day. Overall, the model accounted for 0.069 of the fraction of the variance. Both fearful attachment (β = 0.87, *p* =. 002) and preoccupied attachment (β = 0.84, *p* =. 001) had a positive association with stress reports, while secure (β = −0.083, *p* =. 001) and dismissive attachment (β = −0.088, *p* =. 001) were associated with a negative association with stress reports. Alongside attachment, gender (β = 0.051, *p* =. 029), grade of employment (β = −0.087, *p* =. 001), and age (β = −0.109, *p* =. 001) were also significant predictors.

### Attachment Ratings, Waking Cortisol, and CAR

The attachment styles of secure, preoccupied, fearful, and dismissive did not significantly predict either waking levels of cortisol or CAR when entered into the regression model. The total adjusted *R*^2^ for these models were 0.015 and 0.010, respectively. Employment grade (β = −0.055, *p* =. 046), smoking (β = −0.064, *p* =. 009), and BMI (β = −0.048, *p* =. 049) did predict waking cortisol. CAR was predicted by wake-up time (β = −0.082, *p* =. 001) and smoking (β = 0.064, *p* =. 008) only.

### Attachment Ratings and Cortisol Output Across the Day

**Figure 1:**
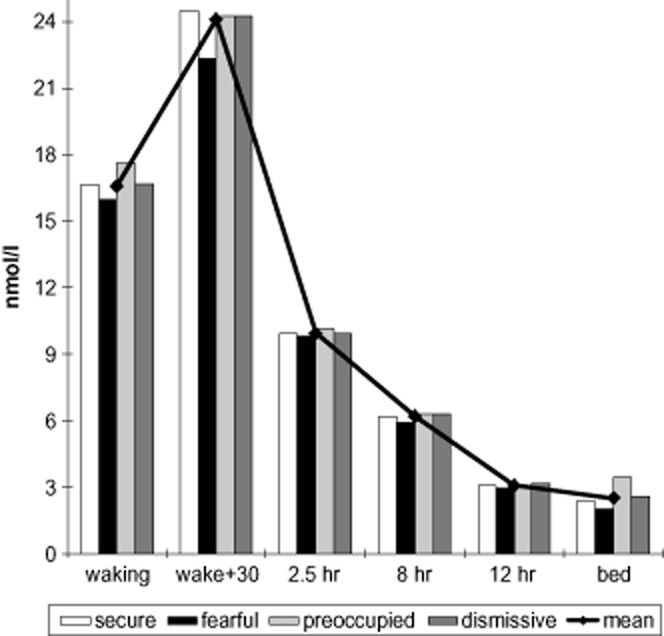
Cortisol profile across the day.

We examined the association between attachment and cortisol across the day by examining area under the curve (AUC_ground_). The model consisted of the four attachment styles and the previously stated covariables. The cortisol profile across the day can be seen in Figure 1. Overall, the model predicted 0.044 of the fraction of the variance. Preoccupied attachment had a significant association with cortisol across the day, while fearful attachment approached significance. Thus, preoccupation ratings were associated with higher levels of cortisol (β = 0.061, *p* =. 017), while fearful attachment ratings had an inverse relationship (β = −0.054, *p* =. 053), indicating a lower output independently of covariates. Mean stress (β = 0.115, *p* =. 001), smoking (β = 0.090, *p* =. 001), gender (β = −0.116, *p* =. 001), and employment grade (β = −0.075, *p* =. 002) were all associated with cortisol over the day.

### Attachment Ratings, Bedtime Cortisol, and Slope of Change of Cortisol Levels Across the Day

The associations between attachment ratings and cortisol were further examined using bedtime values and the slope of the decline of cortisol across the day. Only preoccupied ratings were associated with bedtime cortisol levels (β = 0.096, *p* =. 001). Results indicate that the preoccupied rating was also negatively associated with the slope of decline, indicating a flatter slope (β = −0.052, *p* =. 035). Of the remaining variables, wake-up time also had an inverse association with the slope of decline (β = −0.201, *p* =. 001), whereas mean stress (β = 0.075, *p* =. 002) was positively associated with the slope of decline. Overall, the model predicted 0.064 of the fraction of the variance.

### Gender, Attachment, and Cortisol

Further analyses revealed gender differences in attachment and cortisol response. Although no differences were found in either waking cortisol or CAR (*p* >. 05), differences emerged in cortisol across the day (AUC_ground_). Preoccupied attachment in males was associated with increased cortisol across the day (β = 0.066, *p* =. 021) and bedtime levels of cortisol (β = 0.093, *p* =. 005). Fearful attachment in women saw an inverse association with both cortisol across the day (β = −0.147, *p* =. 015) and bedtime cortisol (β = −0.140, *p* =. 021). No other significant associations were found.

## Discussion

The aims of this study were to investigate the association between attachment and cortisol response over the course of a day in healthy older adults. Contrary to our expectation, no associations were found between attachment and any of the morning cortisol measures (waking and CAR). Little is known regarding the association between attachment and CAR, and the results of previous studies have been inconsistent (Adam & Gunnar, 2001; Quirin et al., 2008). The cortisol rise after awakening may be influenced by anticipation of demands for the upcoming day, with situational factors accounting for up to 60% of variation in CAR response (Hellhammer et al., 2007). Since attachment is a trait characteristic, we would not necessarily therefore expect to find an association with the CAR.

In keeping with the existing literature on acute and diurnal cortisol patterns, preoccupied (high anxiety/low avoidance) attachment was associated with both increased stress perceptions and higher levels of cortisol throughout the day as measured using AUC_ground_ (Quirin et al., 2008). There was also some indication that preoccupied attachment ratings were related to a flatter cortisol profile across the day in the complete sample, because of heightened bedtime cortisol levels. For men in particular, preoccupied attachment was associated with increased day cortisol levels (AUC) and higher bedtime cortisol levels, while only approaching significance among women. Our previous laboratory study had not found any striking pattern of cortisol response to acute stress in the preoccupied group (Kidd et al., 2011). That analysis involved attachment groups only, not regressions involving the full spectrum of preoccupied attachment ratings, and only a small number of individuals had a predominantly preoccupied style.

One tentative explanation for the results obtained for the preoccupied style may be that hypervigilant strategies utilized during waking hours may contribute to an inability to reduce levels of arousal when preparing to sleep, as well as reflecting heightened levels of cortisol through the day (Maunder, Hunter, & Lancee, 2011). The observed pattern of responding suggests that preoccupied attachment may be linked to anticipatory stress appraisals about upcoming events and ineffective strategies for downregulating both subjective and physiological response (Shaver & Mikulincer, 2007). This is supported by the fact that both subjective and physiological levels remained high even until the final measure at bedtime, which may be suggestive of dysregulation, which has been associated with negative health outcomes (Dickerson & Kemeny, 2004).

Contrary to our predictions, fearful attachment was not associated with reduced cortisol across the day in the regressions involving the full range of attachment ratings for the entire sample, although the results approached significance. However, when males and females were examined separately, a significant negative association was found for fearful attachment in women in cortisol across the day and bedtime cortisol. This is despite recording high levels of subjective stress. This may be because fearful attachment is linked to ineffective coping strategies, as being high in attachment avoidance and anxiety means they are unable to achieve any of the goals of anxious and avoidant attachment. As a consequence, the attachment system remains activated due to competing hyperactivating (approach) and deactivating (avoid) strategies.

In addition, the greater age of our population in comparison with previous studies (Quirin et al., 2008) may have had an effect on the HPA axis response to stress events due to cumulative or progressive effects of chronic stress across the life span (Carpenter et al., 2009). Such an interpretation would be consistent with the concept of allostatic load across the life course (McEwen, 2007). There is some evidence to support the idea that chronic activation of the HPA system may initially present as hyperreactivity with fearful attachment in young adulthood (Dewitte et al., 2010; Powers et al., 2006), but over time the HPA axis loses its resilience as a consequence of dysregulation (Kidd et al., 2011).

Dismissive attachment was associated with low stress reports and no association with any of the cortisol parameters. Previous research suggests that those who are high in attachment avoidance disassociate themselves from situations that may threaten autonomy and, as a result, they may experience, or report, less distress (Shaver & Mikulincer, 2007). Contradictory findings reported for dismissive attachment during acute stress may be related to the laboratory situation itself where the use of habitual strategies may not be possible, particularly in interpersonal acute stress tasks (Dewitte et al., 2010; Powers et al., 2006). The results reported in the current work are thought to reflect an average day and so consequently should reflect habitual regulatory responses of dismissive attachment, namely, deactivating strategies. Secure attachment, on the other hand, reflects the ability of the individual to effectively regulate and mitigate the strength of emotional and consequently physiological responses to adverse events.

Both heightened and attenuated cortisol responses are associated with increased morbidity and mortality (Bartley et al., 2007; McEwen, 2007; Newell-Price, Bertagna, Grossman, & Nieman, 2006; Raison & Miller, 2003). Research in the Whitehall study has linked a flatter cortisol profile over the day and elevated evening levels of cortisol with increased cardiovascular mortality (Kumari et al., 2011). Both age and being male were also predictive of this association. Preoccupied attachment, with high overall output and a flatter slope over the day, may be relevant to cardiovascular health outcomes, while fearful attachment with low cortisol output across the day may predict noncardiovascular health outcomes (Fries, Hesse, Hellhammer, & Hellhammer, 2005). This corresponds with the findings of McWilliams and Bailey (2010), who demonstrated an association between anxious attachment and cardiovascular health conditions. Our findings support the hypothesis that susceptibility and response to stress may be one pathway through which attachment may confer health consequences (Maunder & Hunter, 2001).

### Limitations and Future Directions

Caution needs to be taken in interpreting the data presented here as the study was limited to white, middle-aged participants, and so we cannot generalize the results to other age or ethnic groups. In addition, cortisol was sampled over one day only, and it has been recommended that cortisol profiles should be recorded over subsequent days (Hellhammer et al., 2007). It is important to establish the temporal stability of the diurnal cortisol pattern, as a sustained hypo- or hyperreactive response is likely to have more deleterious consequences than if it is transient. Other timing constraints to consider are that cortisol and attachment were measured at different time points, with an approximate 4-year gap. However, the RQ has reported 70% stability of attachment measured over a 4-year period (Hazan & Shaver, 1987).

Furthermore, the RQ has been criticized as a tool for measuring attachment, with dimensional approaches being the gold standard. Although we acknowledge the limitations of this measure, it should be noted that the measures most commonly used today, such as the Experiences in Close Relationships Scale (Brennan et al., 1998), were not available when data was initially collected during Phase 5 of the Whitehall study (1997). Moreover, the RQ is still used in contemporary research for its ease of administration and interpretation. This is particularly true of large-scale studies in which attachment is measured along with many other variables. This is the case in the Whitehall II prospective epidemiological study from which these data were drawn (Bartley et al., 2007). Despite this limitation, the findings provide important preliminary support for the association between attachment and the HPA axis across the day in older adults.

Although our study supports the association between attachment and cortisol, attachment only accounted for a small amount of the variance. Previous research has shown that behavioral and demographic factors, such as smoking and employment grade, are associated with cortisol profiles, as was found in this study (Adam & Kumari, 2009). It is also likely that the physiological response to stress may not be the only mechanism that links attachment to health. There are known associations between attachment and emotional adaptation (Shaver & Mikulincer, 2007), while other work suggests that those who are insecurely attached are less able to seek help and utilize social support effectively, which are also linked to poor health outcomes (Ciechanowski, Walker, Katon, & Russo, 2002; Maunder & Hunter, 2001).

There was no a priori hypothesis concerning gender in the study; however, in accordance with previous work, gender differences were found for attachment and cortisol response (Dewitte et al., 2010; Hicks & Diamond, 2011). Although acute stress studies appear to be more consistent in their findings regarding gender, with a trend of increased cortisol response in anxious males and avoidant females, the findings related to the diurnal cortisol profile have been less robust (Jaremka et al., 2013). Clearly identifying reasons as to why there is such variation is important if we are to understand the potential pathways linking attachment to health.

Our interpretation of the influence of gender is limited for several reasons. Firstly, while there is a record of how stressed participants were feeling when they took their sample, there is no record of the type of stress they were experiencing, if they were alone, or if others were present. This means we are unable to account for factors such as gender norms, which have been shown to contribute to whether a situation is interpreted as being stressful (Pietromonaco, DeBuse, & Powers, 2013). It has also been suggested that effective stress regulation may be dependent in part on the availability and supportiveness of the attachment figure (Pietromonaco et al., 2013). Indeed, preliminary evidence does seem to suggest that dyadic exchange in couples, where one partner is highly anxious and the other avoidant, may result in increased cortisol response during an interpersonal stress task (Dewitte et al., 2010). More research is needed to elucidate how gender and presence of others may interact with attachment and influence the stress response in everyday life.

The results of the current work add to the existing literature as it describes the first large-scale analysis of attachment, subjective stress, and diurnal cortisol response. Our results suggest that attachment may act as a chronic stressor resulting in possible HPA axis dysregulation. Our results may also offer some support regarding possible gender differences in attachment response to stress. Finally, understanding how patterns of diurnal cortisol group together as a function of attachment style may help us to identify “at-risk” groups for increased morbidity and mortality in older populations.
